# Sustainable, Rapid Synthesis of Bright-Luminescent CuInS_2_-ZnS Alloyed Nanocrystals: Multistage Nano-xenotoxicity Assessment and Intravital Fluorescence Bioimaging in Zebrafish-Embryos

**DOI:** 10.1038/srep26078

**Published:** 2016-05-18

**Authors:** S. Shashank Chetty, S. Praneetha, Sandeep Basu, Chetana Sachidanandan, A. Vadivel Murugan

**Affiliations:** 1 Advanced Functional Nanostructured Materials Laboratory, Centre for Nanoscience and Technology, Madanjeet School of Green Energy Technologies, Pondicherry University, Kalapet, Puducherry-605014, India; 2 Council of Scientific and Industrial Research-Institute of Genomics & Integrative Biology (CSIR-IGIB), South Campus, New Delhi 110025, India; 3 Academy of Scientific and Innovative Research (AcSIR), New Delhi 110025, India

## Abstract

Near-infrared (NIR) luminescent CuInS_2_-ZnS alloyed nanocrystals (CIZS-NCs) for highly fluorescence bioimaging have received considerable interest in recent years. Owing, they became a desirable alternative to heavy-metal based-NCs and organic dyes with unique optical properties and low-toxicity for bioimaging and optoelectronic applications. In the present study, bright and robust CIZS-NCs have been synthesized within 5 min, as-high-as 230 °C without requiring any inert-gas atmosphere *via* microwave-solvothermal (MW-ST) method. Subsequently, the *in vitro* and *in vivo* nano-xenotoxicity and cellular uptake of the MUA-functionalized CIZS-NCs were investigated in L929, Vero, MCF7 cell lines and zebrafish-embryos. We observed minimal toxicity and acute teratogenic consequences upto 62.5 μg/ml of the CIZS-NCs in zebrafish-embryos. We also observed spontaneous uptake of the MUA-functionalized CIZS-NCs by 3 dpf older zebrafish-embryos that are evident through bright red fluorescence-emission at a low concentration of 7.8 μg/mL. Hence, we propose that the rapid, low-cost, large-scale “sustainable” MW-ST synthesis of CIZS-NCs, is an ideal bio-nanoprobe with good temporal and spatial resolution for rapid labeling, long-term *in vivo* tracking and intravital-fluorescence-bioimaging (IVBI).

Indeed, intravital fluorescence bioimaging (IVBI) has become an essential research methodology for tracking biomolecules and biological processes in the living organism^1^. Unlike other imaging techniques, fluorescence imaging makes use of low excitation energy in the visible to near-infrared (NIR) spectrum, to visualize across the multi-spatial scale that includes cells, tissue and small animals with high detection sensitivity^2^. Precisely, emission wavelength of 650–900 nm, known as “biological-optical-window” is highly suitable for *in vivo* imaging due to its reduced absorption and scattering process within the living systems^3^. Therefore, IVBI is being widely used as a “workhorse” among the other imaging technologies in the biomedical research^2^. Zebrafish (*Danio rerio*) is one of the fast emerging, imperative, inexpensive animal model because of their genomic and physiological similarities to human beings^4^,^5^. In addition, they are highly suitable for *in vivo* toxicity and bioimaging studies because of their amenability to genetic manipulation and optical transparency^6^,^7^. The well-characterized embryonic development with small size and high permeability to small molecules have made them particularly suitable for rapid high-throughput biochemical screening and surrogates for *in vivo* protein expression, to visualize biological distribution of metabolites and nano-xenotoxicity studies particularly to investigate organ-specific toxicity^7^,^8^,^9^. On the other hand, the “sustainable and bright-luminescent” I-III-VI_2_ inorganic-semiconducting CuInS_2_-based NCs has been proposed for *in vitro* and *in vivo* bioimaging and diagnostic applications^10^,^11^,^12^,^13^. However, a major challenge of pristine CuInS_2_ (CIS) NCs synthesis arises from its ternary compositions^14^. Typically, Cu-S bond is weaker than In-S, that substantially vary the stoichiometry between Cu^+^ and In^3+^ during the NCs synthesis, which results in off-stoichiometry and phase complexity to induce structural defects^15^,^16^. Although various synthesis methods have been adopted^17^,^18^, among them hot-injection method^19^,^20^,^21^ is particularly successful in offering bright luminescent and photostable CIS-NCs. However, they require either longer reaction time at elevated temperatures of 210–230 °C to induce nucleation and/or utilize excess solvent in inert gas atmosphere^14^,^20^. In addition, most of the CIZS-alloyed NCs exhibit quantum yield (QY) of <70% 20,22,23 and lifetime with ambiguous radiative mechanism. These limitations have to be ameliorated by selecting appropriate sustainable-rapid chemical synthesis of high quality alloyed CIZS-NCs for developing state-of-the-art bio-nanoprobe with tunable emissions. Recently, “colloidal chemistry” based non-injection method that uses anion precursor (i.e. 1-dodecanethiol) as both ligand and sulfur source have been well-thought-out, to minimize the requirement of additional solvents during the synthesis^17^,^24^. Pein *et al.* investigated conventional and domestic-microwave heating synthesis of CuInS_2_ nanoparticles using oleylamine as solvent, however the luminescent properties for optical applications were not investigated^25^. Pons *et al.* reported reduced toxicity of CuInS_2_/ZnS NCs, compared to CdTeSe/CdZnS nanocrystals (NCs) for *in vivo* imaging of mice sentinel lymph node^21^. Although nanostructured Ag, CdTe and ZnO, C-dots have been extensively investigated in zebrafish to study toxicity, maldeveloped phenotypes and behavioral testing^26^,^27^,^28^,^29^,^30^, the nano-xenotoxicity and bioimaging applications of CIZS-NCs have never been explored in zebrafish model till date. Recently, we have developed microwave-assisted solvothermal (MW-ST) method to prepare various nanostructured materials for clean energy conversions and storage applications^31^,^32^,^33^,^34^,^35^,^36^,^37^. Here, we demonstrate for the first time, an efficient, hassle-free, highly scalable, one-pot MW-ST synthesis of highly luminescent CIZS-NCs within 5 min at 200 °C and 230 °C, using 1-dodecanethiol (DDT) as both ligand and solvent, without requiring any co-solvents (octadecene, ODE) and inert-gas atmosphere with complete consumption of cationic precursors during NCs synthesis as shown in Fig. 1. Significantly, our efficient MW-ST approach tailored optical band gaps of CIZS-NCs with enhanced fluorescence, emission lifetime (~300 ns) and quantum yield (QY) upto 77% (λ_ex_ ~450 nm) within 5 min synthesis time. The high-quality CIZS-NCs were subsequently phase-transferred into aqueous phase within few minutes *via* surface-ligand exchange technique using 11-mercaptoundecanoic acid (MUA). The *in vitro* analysis in L929, Vero, MCF7 cell lines and multistage *in vivo* nano-xenotoxicity assessment in zebrafish-embryos put forward the versatile application of CIZS-NCs as a biocompatible and robust fluorescent bio-nanoprobe for IVBI in zebrafish model as illustrated in Fig. 1.

## Methods

### Microwave-Solvothermal synthesis of CIZS-NCs

Copper (I) iodide (CuI, Sigma-Aldrich, 99.9%), indium (III) acetate (In(Ac)_3_, Sigma-Aldrich, 99.9%), 1-dodecanethiol (DDT, Sigma-Aldrich, 98%), stearic acid (SA, HiMedia, 95%), zinc chloride (ZnCl_2_, Fisher Scientific, 95%), 11-mercaptoundecanoic acid (MUA, Sigma-Aldrich, 95%) and tetramethylammonium hydroxide pentahydrate (TMAH, HiMedia, 97%) were used without further purification.

**Figure 1:**
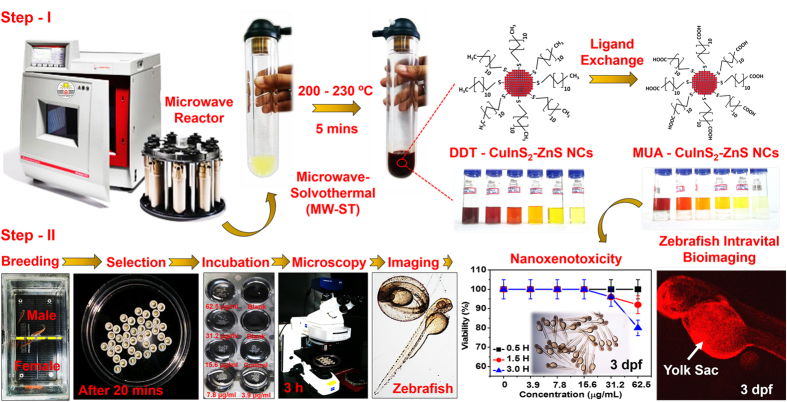
Schematic representation of one-pot MW-ST method to prepare hydrophobic DDT-functionalized CIZS-NCs, subsequently phase transferred into hydrophilic MUA-functionalized CIZS-NCs and systematic protocol followed for nano-xenotoxicity assessment and intravital fluorescence bioimaging application in zebrafish as illustrated in Steps I and II respectively.

In a typical MW-ST synthesis of CIZS-NCs: CuI (0.05 mM), In(Ac)_3_ (0.1 mM), and zinc stearate (0 to 0.1 mM) were mixed in 10 mL of DDT. Preparation of zinc stearate is provided in supplementary information. The reactant mixtures were transferred onto a turnable for uniform heating in Anton-Paar (Multiwave PRO) microwave reaction system SOLV as shown in Fig. 1. The microwave exposure time of 5 min holding at a temperature of 200 °C and 230 °C was programmed with the Anton Paar Multiwave Pro Software. The automatic temperature and pressure control system permitted continuous monitoring of the internal temperature (± 1 °C). The reaction parameters (time, temperature, pressure) were followed automatically by regulating the applied power (0–900 W and pressure upto 80 bar).

The color of the solution changed from white turbid to dark viscous red solution with hypsochromic shift upon increasing zinc precursor concentration. As-synthesized CIZS-NCs solution was further purified by subsequent addition of chloroform, methanol with excess of acetone and precipitated by centrifugation at 10,000 rpm for 10 min. The precipitate was redispersed in 5 mL of chloroform to obtain highly luminescent CIZS-NCs for subsequent phase transformation protocol, as illustrated in Fig. 1.

### Structural and Optical characterization

For structural characterization, X-ray diffraction (XRD) analysis was performed in Rigaku Ultima IV using graphite monochromated Cu Kα radiation (λ = 1.5418 Å) source produced at 40 kV and 30 mA to scan the diffraction angles from 25–80°. The high-resolution transmission electron microscopy (HR-TEM) and selected-area electron diffraction (SAED) patterns was analyzed using a JEOL JEM-4010 electron microscope operated at an accelerating voltage of 400 kV. Energy dispersive X-ray (EDAX) for semi-quantitative elemental analysis was carried out using Thermo SuperDry II. X-ray photoelectron spectroscopy (XPS) measurement was performed using PHOBIOS HSA3500 DLSEGD analyzer with excitation energy of 1486.74 eV. Fourier-transform infrared (FT-IR) analysis was carried out using Thermo Nicolet Model 6700. The zeta (ζ) potential of the MUA-functionalized CIZS-NCs was measured using Zetasizer Nano ZS (Zen 3600, Malvern Instruments). For optical characterization, absorption spectra were recorded in Varian Model 5000. Steady-state photoluminescence (PL) spectra were obtained using FLUOROLOG - FL3-11 (JobinYvon) with excitation wavelength of 450 nm and spectral resolution of 1 nm. Time-resolved photoluminescence (TRPL) measurements were also performed using FLUOROLOG - FL3-11 (JobinYvon) with 488 nm femtosecond laser. The PL QY of samples were measured using rhodamine 6 G (dissolved in ethanol, QY: 95%) as a standard at 450 nm. The QY of CIZS-NCs was calculated using following equation:


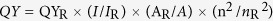


where QY is the quantum yield, I is the integrated PL emission intensity, n is refractive index (n = 1.42 for hexane; n = 1.36 for ethanol) and A is the optical density at the excitation wavelength. The subscript R refers to reference.

### Nano-xenotoxicity assessment and intravital bioimaging

The *in vitro* cell viability (MTT assay) was performed in L929 cell lines (mouse fibroblast cells), MCF7 cell lines (Human breast cancer cells) and Vero cell lines (African green monkey kidney epithelial cells). The cells were cultured in Dulbecco’s modified Eagle’s medium (DMEM), supplemented with 5% heat-inactivated fetal calf serum (FCS) in 5% CO_2_ at 37 °C. After trypsin treatment, 3 × 10^4^ cells/well (100 μl/ml) was seeded into 96-well plate to attain minimal confluency. The cells were treated with different concentration of MUA-functionalized CIZS-NCs and co-incubated for 24 hours. The relative absorbance was recorded at 450 nm to determine cellular viability. For cellular bioimaging studies L929, MCF7 and Vero cells were cultured on the cover slip placed in the 6-well plate and subsequently MUA-functionalized CIZS-NCs (30 μg/mL) were co-incubated for just 20 mins. The MUA-functionalized CIZS-NCs labelled L929 and MCF7 cells on the cover slip were fixed with 4% paraformaldehyde (PFA) and 300 nM 4′,6-Diamidino-2-Phenylindole (DAPI) staining solution. These fixed cells were washed with PBS buffer (5 times) and imaged under inverted fluorescence microscope. The MUA-functionalized CIZS-NCs labeled Vero cells on the cover slip were fixed with 4% PFA and directly imaged under confocal microscope. The *in vivo* nano-xenotoxicity assessment and bioimaging was performed in zebrafish (wild type, Tubingen) embryos. The adult male and female zebrafish (1:1) were placed separately in breeding box maintained with controlled light and temperature conditions: 26 °C with a 14 h/10 h light/dark cycle. Spawning was triggered in the morning, once the light was turned on. The embryos were collected after 20 min and washed three times with E3 medium [5 mM NaCl, 0.17 mM KCl, 0.33 mM CaCl_2_, and 0.33 mM MgSO_4_ (pH 7.2–7.3)] to remove the surrounding debris prior to analysis. The healthy embryos were placed in 12-well culture plates (25 embryos in 2 mL of E3 medium/well) and treated with different concentration of MUA-functionalized CIZS-NCs (0, 3.9, 7.8, 15.6, 31.2, 62.5 μg/mL) on 6 hpf (hours post-fertilization), 24 hpf and 3 dpf (days post-fertilization) zebrafish embryos as illustrated in Fig. 1. All experimental protocols using zebrafish embryos were approved by the Institutional Animal Care Committee of CSIR-IGIB institute, New Delhi, India. The methods were carried out in accordance with the approved guidelines. The embryonic development was assessed using a Carl Zeiss bright-field inverted microscope (Stemi 2000-C) to screen the numbers of viable embryos at different time points for 3 hours in each well. Fluorescence imaging of the 6 hpf, 24 hpf and 3 dpf zebrafish embryos were observed after 0.5 h, 1.5 h and 3 h using a Carl Zeiss Scope A1 microscope attached to a AxioCam HRc detector to show the uptake of co-incubated MUA-functionalized CIZS-NCs for intravital fluorescence bioimaging.

## Result and Discussion

### Structural characterization of CIZS-NCs

**Figure 2:**
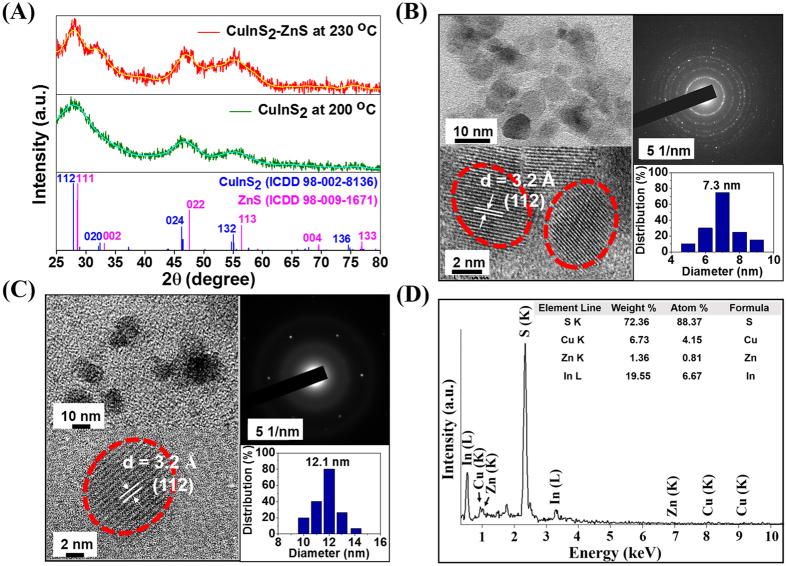
Structural analysis of synthesized CIZS-NCs. (**A**) XRD patterns of MUA-functionalized CIS-NCs and CIZS-NCs synthesized within 5 min *via* MW-ST at 200 °C and 230 °C respectively. HR-TEM images of MUA-functionalized (**B**) CIS-NCs prepared at 200 °C and (**C**) CIZS-NCs prepared at 230 °C. (**D**) EDX spectrum of MUA-functionalized CIZS-NCs at 230 °C.

The X-ray diffraction patterns of chalcopyrite structured I-III-VI_2_ semiconducting CIS and CIS alloyed with ZnS NCs that were synthesized *via* MW-ST at 200 °C and 230 °C respectively have been illustrated in Fig. 2A. The broad diffraction peaks were observed due to the nanoscale structure of CIS and CIZS crystals^10^. While comparing the X-ray diffraction patterns of as-synthesized CIZS-alloyed NCs with standard CuInS_2_ (ICDD No. 98-002-8136) and ZnS (ICDD No. 98-009-1671), three major peaks at 28.2°, 46.8° and 55.4° were well-matched with (112), (024) and (132) planes of tetragonal crystal system respectively and it was slightly shifted to higher angle (2θ) closer to the characteristic peak position of the bulk ZnS. This is obvious due to slight difference in lattice parameters between of zinc blende structure (a = 0.5517 nm) and chalcopyrite structure (a = 0.5345 nm), which is least susceptible to distortion^15^.

**Figure 3:**
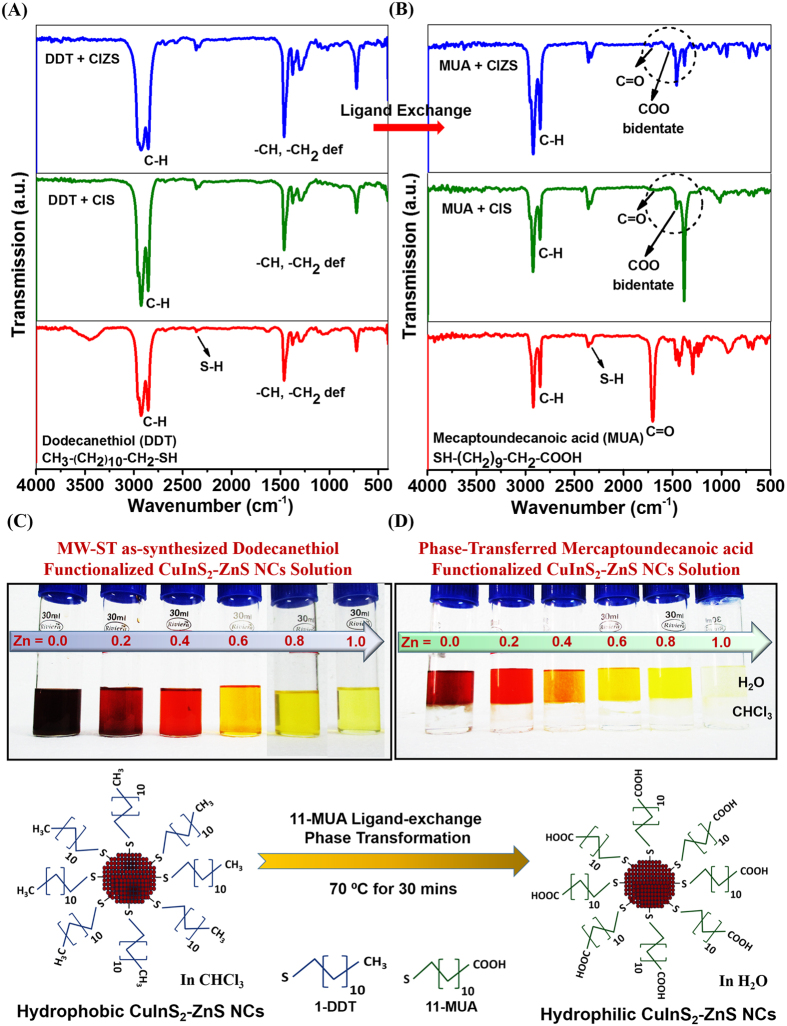
Surface-ligand characterization. FTIR spectra of (**A**) DDT-functionalized CIS-NCs and CIZS-NCs synthesized *via* MW-ST at 200 °C and 230 °C respectively. (**B**) MUA-functionalized CIS-NCs and CIZS-NCs after subsequent ligand exchange. (**C**,**D**) are day-light photographic pictures before and after phase transformation of DDT-functionalized CIZS-NCs.

In addition, the ionic size of zinc, copper and indium have similar probability to be exchanged easily among themselves because of their closer thermodynamic enthalpy of formation (∆H)^38^. Further, HR-TEM analysis of both water soluble MUA-functionalized CIS-NCs prepared at 200 °C and CIZS-NCs prepared at 230 °C exhibited nanocrystalline spherical shape with average particle size distribution of 7.3 ± 2 nm and 12.1 ± 2 nm respectively as shown in Fig. 2B,C. The increase in the particle size could be attributed to cation exchange or alloying of Zn^2+^ with Cu^+^ and In^3+^ ions in CIS-NCs. The clear lattice fringes exhibited with interlayer spacing of 3.2 nm, corresponding to the (112) plane of CIS chalcopyrite structure obtained in XRD analysis. To obtain information about the chemical composition of NCs, energy dispersive X-ray (EDX) analysis was performed. A typical EDX spectrum of Zn^2+^ ions alloying with CIS NCs is shown in Fig. 2D. The EDX spectrum illustrated the presence of indium, deficiency of copper and excess of sulfur due to thiol-rich ligand (DDT) on the surface with alloyed zinc content in the MUA-functionalized CIZS-NCs. It is important to investigate the surface functionalization of the capping ligands that tend to associate with surface of the atoms. In these CIS and CIZS-NCs, we have introduced DDT in the MW-ST synthesis method allowing for the possibilities of DDT-functionalization on the surface of the atoms. The FTIR spectra of MW-ST synthesized hydrophobic DDT-functionalized CIS-NCs and CIZS-NCs at 200 °C and 230 °C respectively are shown in Fig. 3A. The characteristic vibrational mode of DDT-functionalized NCs around 2854, 2875, 2940, and 2960 cm^−1^ are assigned to the symmetric and asymmetric CH_2_ and CH_3_ stretching mode respectively^39^. The peak at 1462 cm^−1^ corresponds to deformation vibration of –CH_2_ and a weak band at 1301 cm^−1^ assigned to C-H stretching vibration, thus confirming the functionalization of DDT ligand on the surface of CIZS-NCs. After hydrophobic to hydrophilic phase transformation, the FTIR spectra of the MUA ligand-exchanged hydrophilic CIS-NCs and CIZS-NCs are shown in Fig. 3B. Since MUA contains the CH_2_ backbone, its spectrum shows both CH_2_ symmetric and asymmetric stretching mode around 2855 and 2940 cm^−1^ respectively. In addition, the FTIR spectrum contains features around 1470 cm^−1^ and bidendate peak at 1722 cm^−1^, which are related to the COOH moieties^35^. Hence, these signatures clearly indicate that MUA displaces DDT preferentially and changes the surface chemistry of the both CIS and CIZS-NCs. Further it was also observed before and after MUA-ligand exchanged reaction in day-light digital photographic pictures as shown in Fig. 3C,D, respectively.

**Figure 4:**
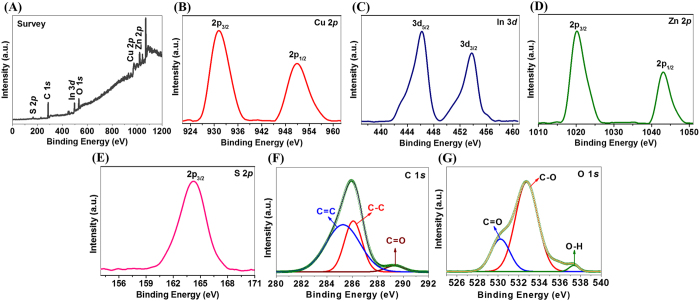
Surface chemical composition analysis of CIZS-NCs. (**A**) XPS survey spectra and high-resolution spectra of (**B**) Cu 2*p*, (**C**) In 3*d*, (**D**) Zn 2*p*, (**E**) S 2*p*, (**F**) C 1*s* and (**G**) O 1*s* of MUA-functionalized CIZS-NCs.

Furthermore, the surface chemistry of phase-transferred MUA-functionalized CIZS-NCs and their oxidation states were investigated by X-ray photoelectron spectroscopy (XPS). The high resolution XPS results from Cu 2*p*, In 3*d*, Zn 2*p*, S 2*p*, C 1*s* and O 1*s* core levels of the MUA-functionalized CIZS-NCs individual elements displayed in Fig. 4A. The core Cu 2*p* in Fig. 4B splits into Cu 2*p*_3/2_ (931.5 eV) and Cu 2*p*_1/2_ (951.2 eV) peaks with standard separation of 19.7 eV. The absence of the satellite peak at 944.0 eV confirmed monovalent Cu^ + ^state. The binding energies (BE) of In 3*d*_5/2_ (444.1 eV) and In 3*d*_3/2_ (451.6 eV) confirmed trivalent In^3+^ state in Fig. 4C. The Zn 2*p*_3/2_ (1021.1 eV) and Zn 2*p*_1/2_ (1044.2 eV) with peak splitting of 23.1 eV confirmed bivalent Zn^2+^ state in Fig. 4D. The S 2*p*_3/2_ (161.4 eV) was also confirmed from XPS analysis assigned to S coordination with Cu, In and Zn in Fig. 4E. As well, the alkane-thiols are reported to be rarely oxidized upon long-time exposure to air (>2 months)^39^. In our present study, MUA-functionalized CIZS-NCs were stored in open and exposed to air for ∼3 months before performing XPS analysis. Interestingly, MUA ligand was much better protected alkane-thiol from aerial oxidation due to the long-chain, hence the S 2*p* feature in our CIZS-NCs sample at BE 167 eV was not present^39^. Deconvolution of the carbon C 1*s* core levels spectra is shown in Fig. 4F, the carbon peak corresponding to the hydrocarbon tail of the MUA-functionalized ligands on the CIZS-NCs is confirmed at 285.2 eV and 286 eV assigned to C = C and C-C respectively. In addition, a higher BE value at 289 eV corresponds to the carboxylate carbon on the CIZS-NCs. The core level oxygen O 1*s* spectra after deconvolution shown in Fig. 4G exhibited two distinct BE features at 530 eV and 532 eV seen on the MUA ligand-exchanged CIZS-NCs due to the carboxylate oxygen which is in good agreement with the FT-IR results. The high BE hump at 535.8 eV is expected most likely due to adsorbed water molecules as reported earlier^39^. Moreover, the zeta (ζ) potential study was also performed to investigate their subsequent potential for *in vivo* applications. The ζ-potential of the MUA-functionalized CIZS-NCs was found to be −48.2 mV due to rich –COOH groups on the surface that further confirmed good stability of colloidal dispersions in water (supporting information Fig. SI-3).

### Steady-state and dynamic room-temperature optical characterization of CIZS NCs

**Figure 5:**
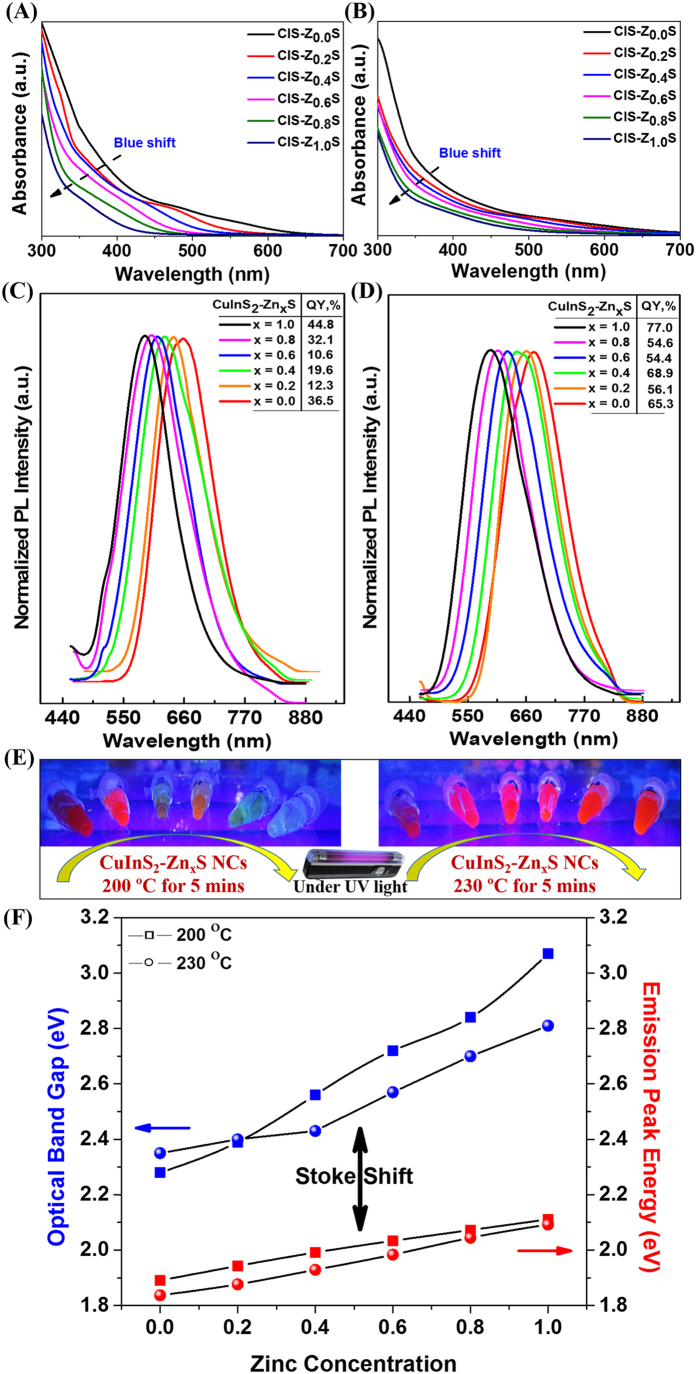
Steady-state room-temperature optical studies of CIZS-NCs. Absorbance spectra of MW-ST synthesized DDT-functionalized CIZS-NCs (**A**) at 200 °C and (**B**) at 230 °C. Photoluminescence spectra of MW-ST synthesized DDT-functionalized CIZS-NCs (**C**) at 200 °C and (**D**) at 230 °C. (**E**) DDT-functionalized CIZS-NCs solutions under UV illumination. (**F**) Comprehensive plot of optical band gap *vs* emission peak energy with increasing zinc concentration (0 to 0.1 mM).

The absorption spectra of as-synthesized DDT-functionalized CIZS-NCs at 200 °C and 230 °C exhibited a broad shoulder below 600 nm, with a trail in the longer wavelength and the absorption was shifted to shorter wavelength with increasing zinc concentration (0 to 0.1 mM) as shown in Fig. 5A,B, respectively. The room-temperature photoluminescence (PL) analysis of DDT-functionalized CIZS-NCs at 200 °C and 230 °C exhibited characteristic blue shift with increasing zinc concentration (0 to 0.1 mM) from 658–589 nm (~70 nm) and 674–591 nm (~83 nm) with full-width at half-maximum (FWHM) of typically ~115 nm as shown in Fig. 5C,D, respectively. The substantially broad emission along with large Stokes shift versus absorption was mainly due to donor-acceptor pair (DAP) recombination^15^,^17^. We measured the quantum yield (QY) of DDT-functionalized CIZS-NCs samples upon increase in zinc concentration from 0 to 0.1 mM, with reference to the standard rhodamine 6G dye (ϕ = 0.95)^15^.

We found that the PL QY of CIZS-QDs was strongly dependent on the reaction temperature under MW-ST condition. While an increase in the reaction temperature from 200 °C to 230 °C, the QY improved significantly from 44.8% to 77% for DDT-functionalized CIZS-QDs. The enhancement of the PL QY by 32.2% at elevated reaction temperature of 230 °C under MW-ST condition is probably due to intrinsic defect-related emissions and off-stoichiometry effects^13^. Hence, DDT-functionalized CIZS-NCs are markedly brighter, robust and exhibit well-defined emission with the position that can be precisely tailored using zinc concentration by using one pot MW-ST method within 5 mins. The comprehensive correlation of optical band gap and their corresponding emission peak energy was analyzed as a function of increasing zinc concentration as shown in Fig. 5E. Hence, we proposed that the possible PL mechanisms were predominantly due to radiative transition through donor-acceptor level or trap sites, instead of band-edge emission due to substantial Stokes shift of 0.54–0.65 eV in DDT-functionalized CIZS NCs at 200 °C and 230 °C. Moreover, these similar results were also recently reported by Chen *et al.*^15^ and Li *et al.*^17^. The donor-acceptor transition mechanism involves In_Cu_ (In substituted at the Cu site) and/or sulfur vacancy (V_S_) as donor and copper vacancy (V_Cu_) as an acceptor^17^. Therefore, it is predicted that DAP recombination between V_S_ donor and V_Cu_ acceptor may be a dominant pathway for tunable NCs emissions, provided the energy levels of the defects centers are flexible with the NCs size. Thus, the incorporated V_Cu_ deficiency in DDT-functionalized CIZS-NCs during MW-ST synthesis is further expected to enhance the PL QY^15^.

**Figure 6:**
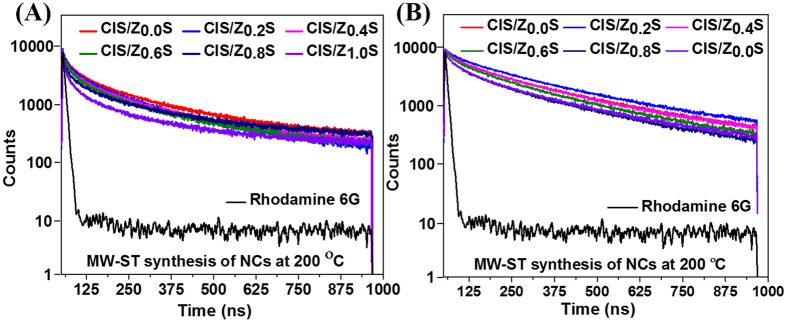
Dynamics luminescence lifetime studies of synthesized CIZS-NCs. (**A**) TRPL exponential decay curve of DDT-functionalized CIZS-NCs at 200 °C with highest lifetime upto ~252 ns and (**B**) exponential decay curve of DDT-functionalized CIZS-NCs at 230 °C with highest lifetime upto ~310 ns.

To further elucidate the emission dynamics of the DDT-functionalized CIZS-NCs, the time-resolved PL lifetime measurements were performed with 488 nm excitation laser as shown in Fig. 6A,B, respectively. The PL decay spectra were tri-exponential in nature and the data was analyzed by least-square iterative statistics. The intrinsic defects, size-dependent band-gaps, and surface defects in the NCs as individual, or in combination are expected to be involved in effective PL emission.

Each of these electron-hole recombination mechanisms correspond to typical PL decay lifetimes. The shorter lifetime (τ_1_) is due to the primarily occupied core-state recombination and longer lifetime (τ_3_) is attributed to the surface-related radiative recombination of carriers. The longest and major lifetime (τ_2_, hundreds of nanoseconds) is due to donor-acceptor transition that accounts for 56–85% photoluminescence in synthesized DDT-functionalized CIZS-NCs. This behavior designate highly emissive recombination channel across the entire NCs communal and is very unusual also rarely observed even in the brightest CdSe NCs^40^. The mean lifetime of DDT-functionalized CIZS-NCs were as high as ~310 ns compared to standard rhodamine 6G dye of 5.5 ns with Chi-square (χ^2^) ≤ 1.1 (supplementary information Fig. SI-2) and Durbin-Watson parameter (DW-P) <2 for fitted parameters as mentioned in Table 1. The 60-fold enhanced lifetime of MW-ST synthesized DDT-functionalized CIZS-NCs compared to organic dye, suggests its excellent long-lived emission with bright photoluminescence characteristics with radiative transition involving an extended electron quantized state and a localized hole state. Based on our PL QY results, the emission lifetime of DDT-functionalized CIZS-NCs at 230 °C was predominantly significant in comparison to heavy-metal based-NCs^40^. Our observation that this behavior would be attributed to large difference in localization volumes of the electron and hole, with a spatial overlap of their wave function that would lead to slower radiative decay. Hence, the enhanced fluorescence and long-lived emission set forth DDT-functionalized CIZS-NCs prepared *via* one-pot MW-ST as a promising optical nanoprobe for extensive time-lapse bioimaging application.

### Nano-xenotoxicity and intravital bioimaging of CIZS-NCs

**Figure 7:**
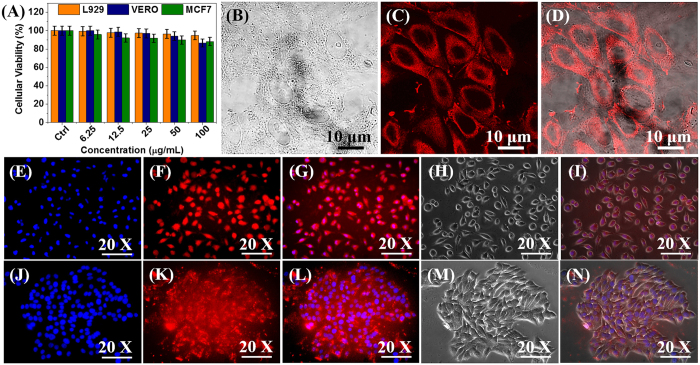
*In vitro* nano-xenotoxicity and cellular bioimaging. (**A**) *In vitro* cellular viability of L929, Vero and MCF7 cell lines treated with varying concentration of MUA-functionalized CIZS-NCs for 24 h. (**B**) Confocal bright-field images, (**C**) MUA-functionalized CIZS-NCs labeled images and (**D**) superimposed images of Vero cell lines. (**E,J**) DAPI-stained images, (**F,K**) MUA-functionalized CIZS-NCs labeled images, (**G,L**) DAPI and MUA-functionalized CIZS-NCs co-labeled images, (**H,M**) phase-contrast images, (**I,N**) superimposed images of L929 and MCF7 cells, respectively.

The *in vitro* cytotoxicity of the MUA-functionalized CIZS-NCs was analyzed using MTT assay on L929, Vero and MCF7 cell lines at different concentration for 24 h. MUA- functionalized CIZS-NCs exhibited substantial cellular viability (≥80%) even at highest concentration (100 μg/ml) as shown in Fig. 7A. Thus we significantly emphasize that MUA- functionalized CIZS-NCs can be considered as a “green” nanoprobe. Recently, Deng *et al.*^2^ reported 9 h co-incubation of high-quality CuInS_2_/ZnS NCs (10 μg/ml) in Bel-7402 and A549 cells for *in vitro* bioimaging applications. On the contrary, we observed “rapid cellular uptake” of our intact MUA- functionalized CIZS-NCs (30 μg/ml) by L929 cells (Fig. 7E–I) and MCF7 cells (Fig. 7J–N) in just 20 mins and further fixing with 4% paraformaldehyde (PFA) with DAPI staining the nuclei as confirmed under inverted fluorescence microscope. Furthermore, the localization of MUA-functionalized CIZS-NCs (30 μg/ml) in the “cellular cytoplasm” was confirmed by labelling Vero cells in just 20 mins and further fixing with 4% PFA for imaging under confocal microscope (Fig. 7B–D). The bright-field (Fig. 7B,H,M) and superimposed fluorescence images (Fig. 7D,I,N) indicated well-defined and bright cellular uptake of MUA-functionalized CIZS-NCs with uniform distribution coupled with bright fluorescence in the cytoplasm of the cells rather than into nuclei. Further specific intracellular localization of MUA- functionalized CIZS-NCs is needed to be investigated using confocal Z-stacks and is still in progress. Owing to high stability, the cells fixed with 4% PFA could be useful for long-term cellular imaging. The intense contrast and ease of preparation of our MUA- functionalized CIZS-NCs together enable us to label both normal (L929, Vero) and cancer (MCF7) cells within just 20 min to visualize cellular morphologies and distribution for intravital bioimaging.

Additionally, the *in vivo* nano-xenotoxicity assessment and IVBI studies of near-infrared emitting MUA-functionalized CIZS-NCs was performed in zebrafish embryos. We performed nano-xenotoxicity studies of MUA-functionalized CIZS-NCs on embryos of different ages to study differential sensitivity on the zebrafish as animal model. Zebrafish eggs were fertilized externally to be staged precisely. We exposed 6 hpf embryos to increasing concentrations of the MUA-functionalized CIZS-NCs for 0.5, 1.5 and 3.0 h and measured their survival.

**Figure 8:**
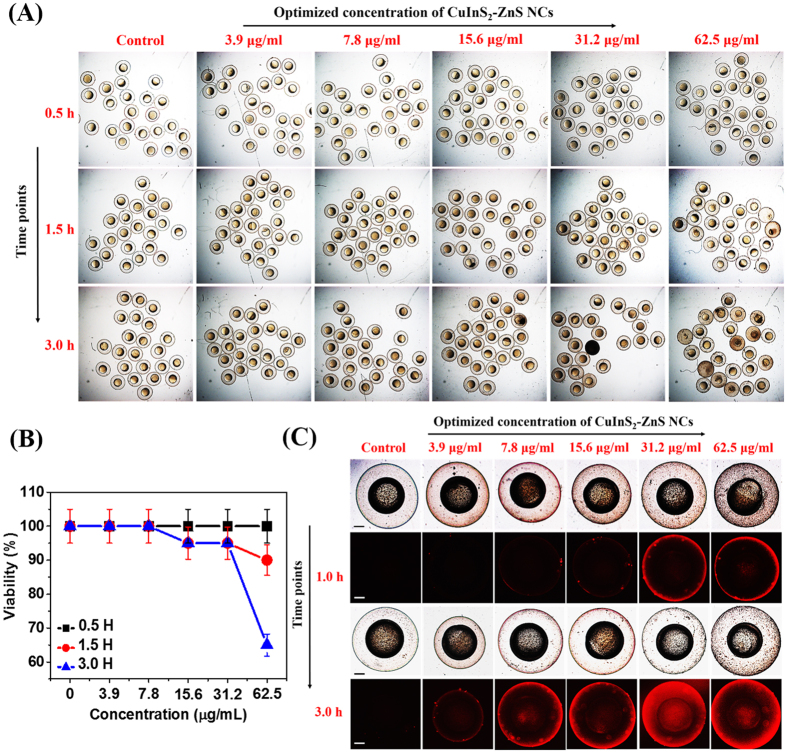
*In vivo* nano-xenotoxicity assessment in 6 hpf zebrafish embryos. (**A**) Bright-field microscopic images at three-time points of 6 hpf zebrafish embryos (N = 25) treated with different concentration MUA-functionalized CIZS-NCs for 3.0 h. (**B**) Embryos viability (%). (**C**) Bright-field (a,c) with fluorescence (b,d) microscopic images at two-time points indicating relative uptake of MUA-functionalized CIZS-NCs in 6 hpf zebrafish embryos.

**Figure 9:**
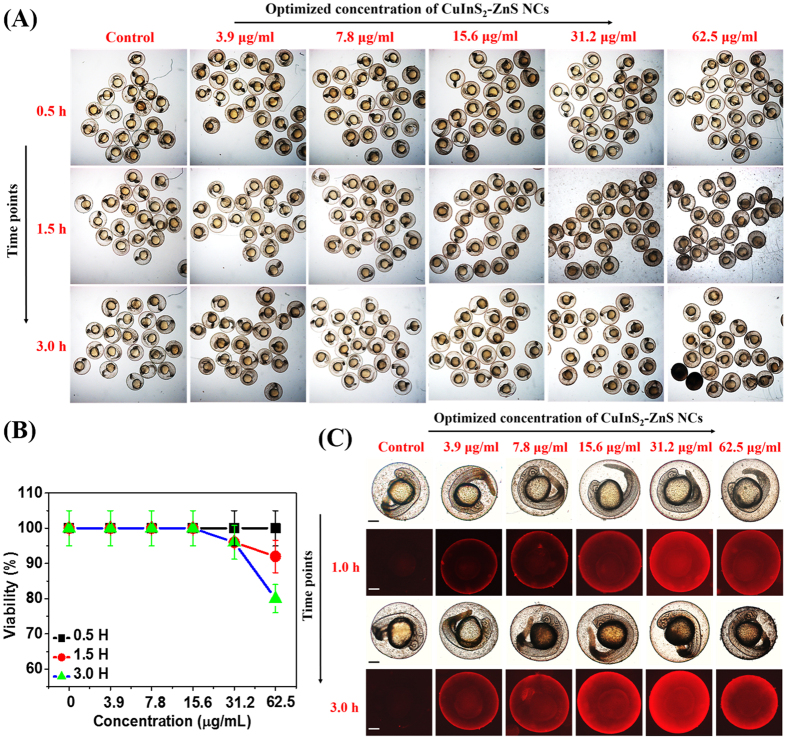
*In vivo* nano-xenotoxicity assessment in 24 hpf zebrafish embryos. (**A**) Bright-field microscopic images at three-time points of 24 hpf zebrafish embryos (N = 25) treated with different concentration MUA-functionalized CIZS-NCs for 3.0 h. (**B**) Embryos viability (%). (**C**) Bright-field (a,c) with fluorescence (b,d) microscopic images at two-time points indicating relative uptake of MUA-functionalized CIZS-NCs in 6 hpf zebrafish embryos.

**Figure 10:**
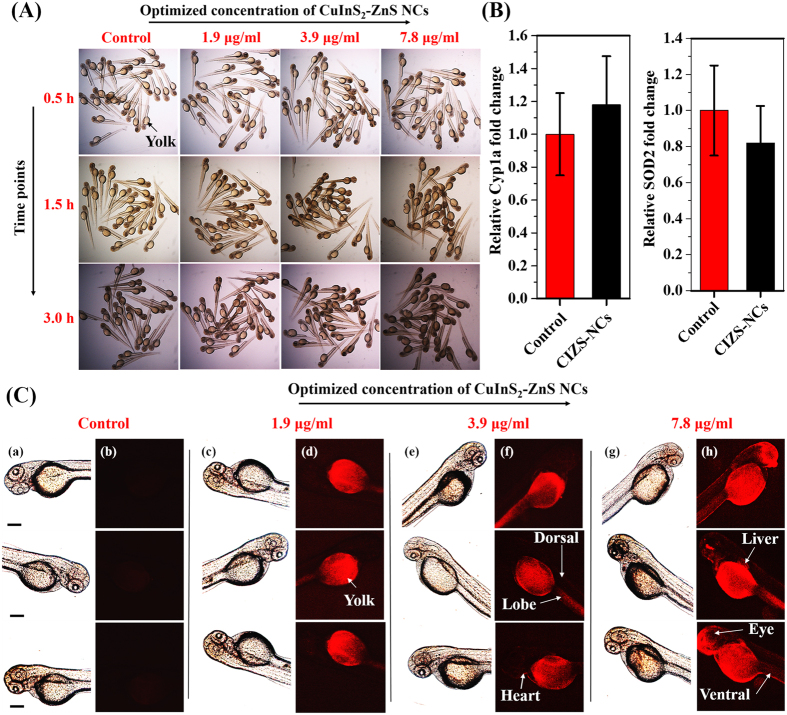
*In vivo* nano-xenotoxicity assessment and intravital imaging in 3 dpf zebrafish embryos. (**A**) Bright-field microscopic images of 3 dpf zebrafish embryos (N = 25) treated with optimized concentrations of MUA-functionalized CIZS-NCs for 3.0 h. (**B**) *CYP1A* and *SOD2* gene expression profile in embryos incubated with 7.8 μg/ml concentration of MUA-functionalized CIZS-NCs. (**C**) Bright-field (a,c,e,g) and fluorescence (b,d,f,h) microscopic images of 3 dpf zebrafish embryos co-incubated with optimized concentrations of MUA-functionalized CIZS-NCs at 3.0 h.

We found that almost all the embryos survived in lower concentrations and at highest concentration tested *i.e.* at 62.5 μg/ml, >60% of embryos retained their viability as shown in Fig. 8A,B. The zebrafish embryos grew within a transparent membrane, the chorion for the first two days. Moreover, we observed bright red fluorescence on the surface of the chorion of live embryos after 1.5 h co-incubation with 31.2 μg/ml MUA-functionalized CIZS-NCs as shown in Fig. 8C and subsequently visualized inside the chorion after 3 h of co-incubation with 7.8 μg/ml MUA-functionalized CIZS-NCs in comparison with the control untreated embryos. The transparent chorions have pores size ranging from 500 to 700 nm and the ~10 nm MUA-functionalized CIZS-NCs can easily diffuse through chorion into the intrachorionic space. The exposure of nanomaterials (Ag^26^, CdTe^27^, ZnO^28^, graphene^41^, TiO_2_^42^ etc.) have been reported to adversely affect the early embryonic development of zebrafish, exhibiting malformed heads, cardiovascular issue, stunted growth, axial malformation and edema^9^,^43^. We also investigated the toxicological effects of MUA-functionalized CIZS-NCs on older embryos. We exposed 1-day old zebrafish embryos to different concentration of MUA-functionalized CIZS-NCs for 0.5, 1.5 and 3.0 h. We found significant viability (>80%) even at the highest concentration tested *i.e.* at 62.5 μg/ml as shown in Fig. 9A,B. The nano-xenotoxicity assessment of MUA-functionalized CIZS-NCs showed that the viability of 24 hpf embryos improved significantly over the 6 hpf embryos. The embryos exhibited regular heartbeats, blood-flow and tail movements under the microscope. We also found that short-term exposures for 3 h did not cause any significant acute teratogenic effects on the zebrafish embryos. The concentration and time-dependent internalization of MUA-functionalized CIZS-NCs through the chorion upon simply soaking the embryos, was evident in 1-day old embryos also through bright-red fluorescence in the yolk sac as shown in Fig. 9C. Younger embryos grew inside the chorion until about 2–3 days, when they spontaneously hatched out and became free swimming. The absence of chorion also changes the sensitivity to drugs and nanoparticles. We tested the effect of exposure to the MUA-functionalized CIZS-NCs on 3-days old embryos. The hatched and free swimming 3-days old embryos were incubated with different concentrations of the MUA-functionalized CIZS-NCs for 0.5, 1.5 and 3.0 h. We observed 100% viability at the optimum concentration tested at 7.8 μg/ml for 3.0 h as shown in Fig. 10A. Embryos exposed to nanoparticles/NCs have been shown to induce cytochromeP450 (cyp) and the superoxide dismutase (SOD2) gene expression in response to xenotoxic stress^44^.

Although we observed no overt signs of toxicity, we tested for the gene expression of *CYP1A* and *SOD2* using real-time quantitative polymerase chain reaction (qRT-PCR) in the embryos incubated with MUA-functionalized CIZS-NCs for 3 h as described in supporting informations.

We found that there was no significant change in the expression of these genes in the MUA-functionalized CIZS-NCs treated zebrafish embryos. The primers used for the qRT-PCR experiments are mentioned in Table 2.

In the absence of chorion, the 3-days old embryos are more exposed to the MUA-functionalized CIZS-NCs, so we examined the uptake of the NCs by the embryos. We found significant increase in fluorescence in the embryos incubated for 3 h in even the lowest concentration tested *i.e*. at 1.9 μg/ml. The fluorescence was predominantly in the yolk at this concentration. However, in 7.8 μg/ml concentration we were able to observe the fluorescence in the head, skin and in the region of the liver indicating rapid uptake of the MUA-functionalized CIZS-NCs by the embryos. These findings substantiate that MW-ST prepared CIZS-NCs are benign with bright fluorescence and long-lived emission in biological media for *in vivo* intravital bioimaging.

## Conclusion

In summary, we have reported a new, efficient, hassle-free and highly scalable one-pot microwave–solvothermal (MW-ST) synthesis of bright luminescent CIZS-NCs within 5 min at 200 °C and 230 °C, using 1-dodecanethiol (DDT) as both ligand and solvent without requiring any co-solvent and/or inert-gas atmosphere. The approach tailored optical band-gaps of “sustainable CIZS-NCs” with enhanced fluorescence and long-lived emission lifetime (~310 ns) with significant quantum yield (QY) upto 77%, without shell-coating strategies. The Cu-deficiency brought about enhanced internal-defect related emission than surface-traps. The ensemble of the results provide an accessible and straightforward labeling platform for rapid visualization of MUA-functionalized CIZS-NCs explicitly in the cellular cytoplasm within 20 min and zebrafish embryos in 3 h for myriad of intravital fluorescence bioimaging investigations.

## Additional Information

**How to cite this article**: Chetty, S. S. *et al.* Sustainable, Rapid Synthesis of Bright-Luminescent CuInS_2_-ZnS Alloyed Nanocrystals: Multistage Nano-xenotoxicity Assessment and Intravital Fluorescence Bioimaging in Zebrafish-Embryos. *Sci. Rep.* **6**, 26078; doi: 10.1038/srep26078 (2016).

## Supplementary Material

Supplementary Information

## Figures and Tables

**Table 1:** Fitting parameters of PL dynamics of CIZS-NCs at 200 °C and 230 °C.

**NC 230 °C**	**A**_**1**_ **(%)**	**τ**_**1**_ **(ns)**	**A**_**2**_ **(%)**	**τ**_**2**_ **(ns)**	**A**_**3**_ **(%)**	**τ**_**3**_ **(ns)**	**χ**^**2**^	**DW-P**	**τ**_**avg**_ **(ns)**
CIS-Z_0.0_S	14.17	88.99	83.07	307.99	2.76	21.53	1.1	1.8107	297.1
CIS-Z_0.2_S	18.46	129.93	79.5	337.73	2.04	27.12	1	1.8236	309.5
CIS-Z_0.4_S	13.78	89.46	83.92	284.19	2.31	20.79	1.1	1.8071	274.1
CIS-Z_0.6_S	17.5	94.07	80.09	292.84	2.4	19.68	1.1	1.7714	279.3
CIS-Z_0.8_S	18.07	80.17	78.36	281.94	3.57	19.73	0.9	1.8387	268.7
CIS-Z_1.0_S	16.58	68.51	79.78	282.86	3.64	15.26	1	1.8959	271.9
**NC 200 °C**	**B**_**1**_ **(%)**	**τ**_**1**_ **(ns)**	**B**_**2**_ **(%)**	**τ**_**2**_ **(ns)**	**B**_**3**_ **(%)**	**τ**_**3**_ **(ns)**	**χ**^**2**^	**DW-P**	**τ**_**avg**_ **(ns)**
CIS-Z_0.0_S	20.57	61.28	68.71	250.37	10.72	15.08	1.1	1.9278	235.5
CIS-Z_0.2_S	22.87	66.5	65.67	247.53	11.46	15.37	1	1.9886	229.9
CIS-Z_0.4_S	24.35	68.15	61.93	257.05	13.72	15.86	1.1	1.857	236.4
CIS-Z_0.6_S	22.48	66.77	71.83	260.57	5.7	15.7	1.1	1.9677	245.1
CIS-Z_0.8_S	20.06	58.59	75.88	263.64	4.06	13.35	1.1	1.9314	251.6
CIS-Z_1.0_S	23.64	57.48	56.29	238.12	20.07	14.62	1	1.9226	217.46

**Table 2:** Primer sequences for RT-PCR Quantification.

Name	Oligonucleotide Sequence
dr-SOD2-F	5′AAGTCTCCCTTCAGCCTGCAT3′
dr-SOD2-R	5′TGAAATGAGCCAAAGTCACGC 3′
dr-CYP1A-F1	5′ AACCAGTGGCAAGTCAACCA 3′
dr-CYP1A-R1	5′ TTCAGTTCAGTACCGTCCGC 3′
zRPL-F	5′TCTGGAGGACTGTAAGAGGTATGC 3′
zRPL-R	5′ AGACGCACAATCTTGAGAGCAG3′
