# Corrigendum: Sustainable, Rapid Synthesis of Bright-Luminescent CuInS_2_-ZnS Alloyed Nanocrystals: Multistage Nano-xenotoxicity Assessment and Intravital Fluorescence Bioimaging in Zebrafish-Embryos

**DOI:** 10.1038/srep28607

**Published:** 2016-06-27

**Authors:** S. Shashank Chetty, S. Praneetha, Sandeep Basu, Chetana Sachidanandan, A. Vadivel Murugan

Scientific Reports 6: Article number: 26078; 10.1038/srep26078 published online: 05182016; updated: 07272016.

The Acknowledgements section in this Article is incomplete.

“This work was financially supported by the Department of Science and Technology, Technology system development (DST-TSD) grant no. PT/2011/178-G, Government of India, New Delhi under “Development of cadmium free high-luminescence III-V and I-III-VI semiconductors based core-shell nanocrystals as nanoprobes for bioimaging applications” for providing the microwave-assisted synthesis facility in our lab. We also thank Dr. Shamima Hussain, UGC-DAE, India for XPS analysis and the Central Instrumentation Facilities (CIF), Pondicherry University for optical and FTIR analysis”.

should read:

“This work was financially supported by the Department of Science and Technology, Technology system development (DST-TSD) grant no. PT/2011/178-G, Government of India, New Delhi under “Development of cadmium free high-luminescence III-V and I-III-VI semiconductors based core-shell nanocrystals as nanoprobes for bioimaging applications” for providing the microwave-assisted synthesis facility in our lab. We thank Dr. V. Ganesan and Dr. Shamima Hussain, UGC-DAE, India for XPS analysis and the Central Instrumentation Facilities (CIF), Pondicherry University for optical and FTIR analysis. We also thank the help from Dr. R. S. Jayasree, and her student S. Hema, Biophotonics & Imaging Lab, SCTIMST, DST-Unit, Trivandrum, India for optimizing *in vitro* studies in L929 and MCF7 cell lines using our CIZS nanocrystals . We also thank the help from Dr. G. Dhinakar Raj and Mr. T.M. Chozhavel, TRPVB, Madhavaram, Chennai, India for confocal imaging of our CIZS nanocrystals labelled VERO cell lines”.

