# Supplementary material for: Sustainable, Rapid Synthesis of Bright-Luminescent CuInS2-ZnS Alloyed Nanocrystals: Multistage Nano-xenotoxicity Assessment and Intravital Fluorescence Bioimaging in Zebrafish-Embryos
Source: Sci Rep. 2016 May 18;6:26078. doi: 10.1038/srep26078 (PMC4870560; doi:10.1038/srep26078)
Supplement: Supplementary Information [file srep26078-s1.pdf]

Supplementary information

Sustainable, Rapid Synthesis of Bright-Luminescent $CuInS_2$-ZnS Alloyed Nanocrystals: Multistage Nano-xenotoxicity Assessment and Intravital Fluorescence Bioimaging in Zebrafish-Embryos

S. Shashank Chetty[a], S. Praneetha[a], Sandeep Basu[bc], Chetana Sachidanandan[bc] and A. Vadivel Murugan[a]*,

[a]*Advanced Functional Nanostructured Materials Laboratory, Centre for Nanoscience and Technology, Madanjeet School of Green Energy Technologies, Pondicherry University, (A Central University), Dr. R. V. Nagar, Kalapet, Puducherry-605014, India.*

[b]*Council of Scientific and Industrial Research-Institute of Genomics & Integrative Biology (CSIR-IGIB), South Campus, New Delhi 110025, India*

[c]*Academy of Scientific and Innovative Research (AcSIR), New Delhi*

*Tel. +91-413-2654975, *E-mail:avmrajeshwar@gmail.com; avmurugan.nst@pondiuni.edu.in.*

Preparation of Zinc Stearate[1]. Briefly, 10 mM SA was dissolved in 20 mL of methanol by heating at 50 °C to obtain a clear solution. To the above solution, 16 mM of TMAH in 5 mL of methanol was added dropwise and stirred for 20 mins. Further, 5 mM $ZnCl_2$ in 10 mL of methanol was added dropwise under vigorous stirring to obtain white precipitate solution. This solution was centrifuged, washed and vacuum dried to obtain pristine zinc stearate.

Phase transformation of Oil soluble CIZS-NCs[2]. Water soluble CIZS-NCs was prepared by exchanging hydrophobic ligand (DDT) with hydrophilic ligand (MUA). 0.5 g MUA was dissolved in 5 mL of methanol and adjusted to pH 9 using TMAH. 0.5 mL of above MUA-methanol solution was added to purified 5 mL of CIZS-NCs in chloroform and stirred for 30 min at 70 °C. Further, 5 mL of distilled water was added and kept stirring for another 20 min. Finally,

the complete solution was separated into two phases (organic/aqueous) with CIZS-NCs phase-transferred into supernatant aqueous phase. The water-soluble MUA-capped CIZS-NCs were collected and stored at room-temperature.

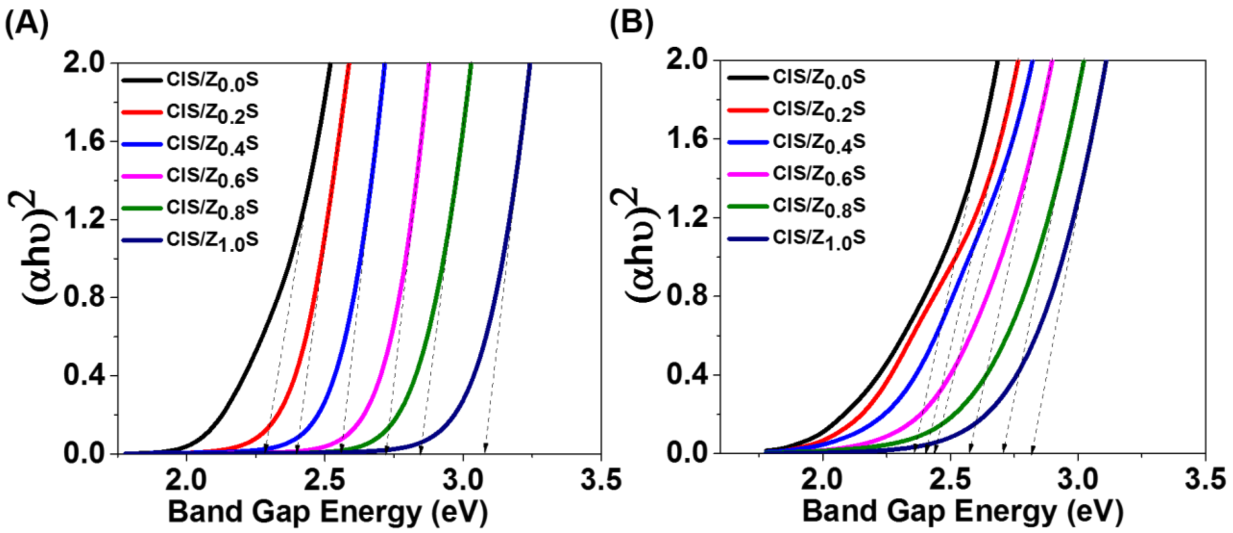

Figure SI-1 | Tauc plot. Zinc concentration dependent optical bandgap of MW-ST synthesized CIZS-NCs at (a) 200 ºC and (b) 230 ºC

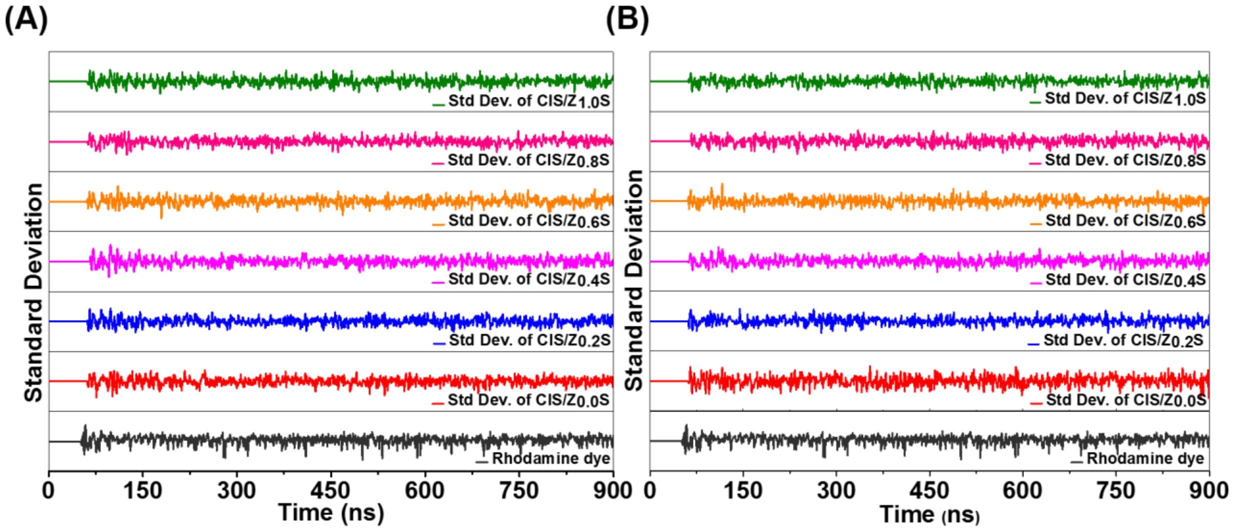

Figure SI-2 | Standard deviation plot of tri-exponential fitting. Shows dynamic PL exponential decay curve of synthesized CIZS-NCs at (A) 200 ºC and (B) 230 ºC.

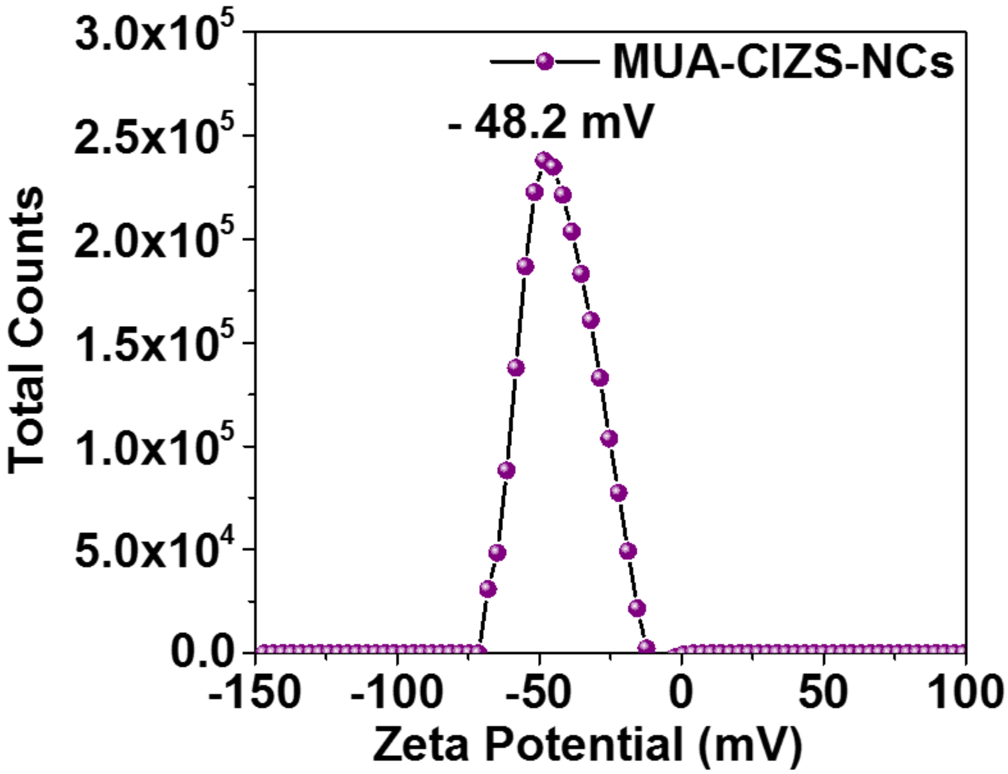

Figure SI-3 | Zeta (ζ) potential analysis. Shows Zeta potential spectrum of phase-transferred MUA-functionalized CIZS-NCs.

Real-time quantitative PCR. Six biological replicate pools of zebrafish embryos (each pool, N=20) were used for each sample. RNA was isolated using RNA isoplus (Takara)[3]. Purity was assessed by 260/280 nm absorbance ratios in Nanodrop1000 (Thermo scientific) and quality was monitored by agarose gel electrophoresis. On average 1µg RNA was used for cDNA synthesis. One control cDNAs was subjected to PCR amplification with serial dilutions to check the amplification efficiency of primers used in qRTPCR. cDNA was synthesized by using QuantiTect Reverse Transcription kit (Qiagen) and realtime quantitative PCR was performed by using sybr green chemistry (Roche) in Light cycler-480 (Roche) platform. The fold change with respect to control was calculated by the $2^{-\Delta\Delta CT}$ algorithm (known as the delta-delta-Ct or ddCt algorithm)[4]. The data were normalized to the endogenous control 18s ribosomal protein small (RPL113 α) gene. Melting and amplification curve analysis was also performed to check the specificity of product.

Reference.

1. Jana, S. *et al.* Prevention of Photooxidation in Blue-Green Emitting Cu doped ZnSe Nanocrystals. *Chem Comm. (2010).*

2. Zhang, W. *et al*. Design and Synthesis of Highly Luminescent Near-Infrared-Emitting Water-Soluble CdTe/CdSe/ZnS Core/Shell/Shell Quantum Dots. *Inorg. Chem.* 48, 9723-9731 (2009).

3. Peterson, S.M. and Freeman, J.L. RNA Isolation from Embryonic Zebrafish and cDNA Synthesis for Gene Expression Analysis. *Journal of Visualized Experiments : JoVE*. 30, 1470 (2009).

4. Livak, K. J. and Schmittgen, T. D. Analysis of relative gene expression data using real-time quantitative PCR and the 2(T)(-Delta Delta C) method. *Methods*, 25, 402-408. (2001)
